# Antimicrobial resistance patterns in a Qatari tertiary care center in 2024: A retrospective study

**DOI:** 10.5339/qmj.2026.52

**Published:** 2026-08-18

**Authors:** Bassem Al Hariri, Ahmad Eid Nazzal Alharafsheh, Joudi Alhariri, Ali Mousa Alnaimat, Manish Barman, Muad Abdi Hassan, Hatem Mabrouk Taher Abusriwil

**Affiliations:** 1 Department of Medicine, Hazm Mebaireek General Hospital, Hamad Medical Corporation, Doha, Qatar; 2 College of Medicine, Weill Cornell Medicine, Doha, Qatar; 3 College of Medicine, Qatar University, Doha, Qatar; 4 College of Medicine, Syrian Private University, Damascus, Syria

**Keywords:** Antimicrobial resistance, antimicrobial stewardship, tertiary care center, multidrug-resistant organisms, infection control, Qatar

## Abstract

**Background:**

Antimicrobial resistance (AMR) is a critical global health threat. Local, real-time epidemiological data are the cornerstone of effective antimicrobial stewardship. This study aimed to establish a 2024 baseline for AMR patterns at a major tertiary care center in Qatar to directly guide empirical therapy and stewardship interventions.

**Methods:**

We conducted a retrospective, cross-sectional analysis of 1,656 positive bacterial cultures from blood, urine, and wound samples collected from 1,218 patients between January 1 and December 31, 2024. Antibiotic susceptibility testing was interpreted per European Committee on Antimicrobial Susceptibility Testing (EUCAST) 2024 guidelines. Statistical analysis utilized descriptive statistics and Chi-square tests.

**Results:**

The most common pathogens were Escherichia coli (30.0%), Staphylococcus aureus (25.0%), and Pseudomonas aeruginosa (15.0%). High resistance rates were observed, with E. coli exhibiting resistance to ceftriaxone (45.0%, 95% CI 40.5–49.5) and ciprofloxacin (50.0%, 95% CI 45.5–54.5). Methicillin-resistant S. aureus (MRSA) prevalence was 30.0% (95% CI 25.6–34.7). Carbapenem-resistant Klebsiella pneumoniae (CRKP) accounted for 5.5% (95% CI 4.4–6.7) of all isolates. Multidrug-resistant organisms (MDROs) were identified in 35.0% (95% CI 32.7–37.3) of samples, with a significantly higher prevalence in intensive care units (ICUs) (45.0%) compared to other wards (p < 0.001).

**Conclusion:**

This study documents a high burden of AMR, dominated by Gram-negative pathogens in critical care settings. These findings necessitate a multi-pronged response, including the revision of empirical antibiotic guidelines, enhanced stewardship focused on high-risk agents, and intensified infection control in ICUs. Integrating molecular epidemiology into future prospective surveillance is essential to combat this escalating crisis.

## 1. INTRODUCTION

Antimicrobial resistance (AMR) represents a significant global health crisis, systematically undermining the efficacy of antibiotics and jeopardizing modern medical practices. The 2022 GRAM report quantified this burden, attributing 1.27 million deaths directly to bacterial AMR in 2019.^1^ Within the Middle East and North Africa (MENA) region, recent estimates suggest that AMR was associated with approximately 256,000 deaths in 2019, with carbapenem-resistant Acinetobacter baumannii and Klebsiella pneumoniae among the leading contributors.^2,3^ In Qatar, while comprehensive mortality data are limited, studies have documented high carriage rates of resistant organisms and increasing trends in clinical infections.^4,5^ The MENA region is a recognized hotspot for AMR, with high rates of resistance in both Gram-negative and Gram-positive pathogens, often exacerbated by substantial antibiotic consumption and challenges in infection control implementation.^2,3^

Qatar, a high-income nation within the MENA region, is not exempt from this threat.^4^ While high-income countries often have robust healthcare infrastructure, they also face unique AMR drivers including high consumption of broad-spectrum antibiotics, substantial international travel and workforce mobility, and concentration of tertiary care services that manage complex, often previously treated patients.^5^ These factors can contribute to the introduction and amplification of resistant strains. Recent national and institutional studies have highlighted concerning trends, including the impact of the COVID-19 pandemic on AMR.^5^ However, real-time, hospital-level data from the most recent complete year to guide empirical therapy at our center were lacking. The rise of multidrug-resistant organisms (MDROs), particularly carbapenem-resistant Enterobacteriaceae, poses a major challenge for tertiary care facilities managing complex patient populations.

Effective empirical therapy and robust antimicrobial stewardship programs are predicated on a precise, contemporary understanding of local resistance patterns. To address this critical gap, this study provides a comprehensive, high-volume, single-year analysis of AMR trends at Hamad General Hospital (HGH), Doha, Qatar, in 2024. Our primary objective was to determine the prevalence and distribution of key bacterial pathogens and their resistance profiles. Blood, urine, and wound infections represent a majority of clinically significant bacterial infections in our setting, and the intensive care unit (ICU) is a known epicenter for AMR emergence, making comparative analysis crucial. Carbapenem-resistant Klebsiella pneumoniae (CRKP) was selected as a specific secondary objective because: (1) it is a leading cause of healthcare-associated infections in our ICUs; (2) carbapenems are last-line antibiotics for severe Gram-negative infections; (3) regional surveillance data have indicated emerging CRKP outbreaks in Gulf Cooperation Council (GCC) countries; and (4) CRKP serves as a key indicator organism for the effectiveness of infection control and stewardship.^5^ Secondary objectives included assessing the burden of MDROs and CRKP and comparing resistance rates between critical care and non-critical care units.

## 2. MATERIALS AND METHODS

### 2.1. Study design and setting

This retrospective, cross-sectional study was conducted at HGH, Doha, Qatar, a major tertiary care center in Qatar. The study was approved by the Institutional Review Board of the Medical Research Center at Hamad Medical Corporation (Approval number: MRC-01-25-331). The requirement for informed consent was waived due to the retrospective design. Ethics approval and consent to participate are detailed in the supplementary materials.

### 2.2. Data collection and definitions

The study population included all patients with positive cultures from blood, urine, or wound specimens that met Centers for Disease Control and Prevention (CDC) criteria for confirmed infection.^6^ Confirmed infectious episodes were defined as positive cultures from specimens collected based on clinical suspicion of infection, accompanied by corresponding antimicrobial treatment as documented in the electronic medical record (EMR). Inclusion criteria required complete antibiotic susceptibility testing (AST) results. We excluded cultures with no growth, contamination, mixed flora not meeting infection criteria, isolates representing colonization, and all fungal isolates. A total of 37 samples were excluded (15 for missing data, 22 for contamination), yielding a final analytical sample of 1,656 confirmed infectious episodes from 1,218 patients. For patients with multiple positive cultures, only the first isolation per patient per pathogen was included in the resistance proportion of calculations to avoid over-representation. However, all unique infectious episodes from different dates or specimen types were counted in the overall sample and MDRO prevalence.

Data were systematically extracted from EMR and the laboratory information management system. Collected variables included age, sex, hospital unit, specimen type, isolated pathogen(s), and AST results. Bacterial identification and AST were performed using the VITEK® 2 automated system (bioMérieux, France). The clinical microbiology laboratory operates under international accreditation (ISO 15189) and participates in external quality assurance programs. Results were interpreted per European Committee on Antimicrobial Susceptibility Testing (EUCAST) clinical breakpoints, version 2024.^7^ Isolates were considered pathogenic (infection) rather than colonizing if they were recovered from sterile sites (blood) or non-sterile sites with clinical signs (e.g., pyuria, purulent discharge) and resulted in a therapeutic intervention. MDROs were defined as isolates exhibiting non-susceptibility to ≥1 agents in ≥3 antimicrobial categories.^8^

### 2.3. Statistical Analysis

Data were analyzed using IBM SPSS (Statistical Package for the Social Sciences) Statistics Version 26.0. Descriptive statistics (means, standard deviations, medians, proportions with 95% confidence intervals [95% CI]) summarized the data. Categorical variables (e.g., MDRO prevalence by unit) were compared using the Chi-square or Fisher’s exact test. A p-value < 0.05 was considered statistically significant. Missing data were handled by listwise deletion. Given the descriptive, cross-sectional aim of establishing a baseline prevalence, the analysis was primarily descriptive. Comparative analyses (e.g., intensive care unit [ICU] vs. non-ICU) utilized Chi-square tests. Multivariate modeling, while possible with the dataset, is reserved for future prospective analyses focusing on risk factors. As a retrospective study aiming to describe all eligible cases within a defined period, a formal sample size calculation was not performed. The study period was chosen to capture a full, recent calendar year (2024) to establish a contemporary baseline.

## 3. RESULTS

### 3.1. Demographics and specimen distribution

A total of 1,218 patients with 1,656 confirmed infectious episodes were analyzed. The mean age was 52 years (±16.5 SD), and the cohort was male (78.0%). The high male predominance reflects the demographic profile of the expatriate workforce receiving care at our center. Specimen distribution was urine (45.0%), blood (30.0%), and wound swabs (25.0%) (Table 1).

### 3.2. Pathogen distribution

The predominant pathogens were Escherichia coli (30.0%), Staphylococcus aureus (25.0%), Pseudomonas aeruginosa (15.0%), and Klebsiella pneumoniae (10.0%). Pathogen distribution varied significantly by source: E. coli dominated urine cultures (55.0%), while S. aureus was most common in wound swabs (40.1%) (Table 2).

### 3.3. Antimicrobial resistance patterns and MDRO prevalence

High resistance rates to first-line agents were observed (Table 3). E. coli resistance was 45.0% (95% CI 40.5–49.5) to ceftriaxone and 50.0% (95% CI 45.5–54.5) to ciprofloxacin. MRSA prevalence was 30.0% (95% CI 25.6–34.7). Notably, CRKP accounted for 5.5% (95% CI 4.4–6.7) of all isolates. ICU-specific resistance was notably high, with 55% of K. pneumoniae isolates being meropenem-resistant, and 60% of P. aeruginosa isolates being ceftazidime-resistant.

The overall MDRO prevalence was 35.0% (95% CI 32.7–37.3; 580/1656), with a significantly higher burden in ICUs (45.0%, 95% CI 39.5–50.5) compared to medical wards (25.0%, 95% CI 21.0–29.0) and the emergency department (20.0%, 95% CI 16.5–23.5) (p < 0.001). All tested CRKP isolates remained susceptible to colistin.

## 4. DISCUSSION

This 2024 baseline analysis documents a high prevalence of AMR at our tertiary care institution in Qatar, with Gram-negative pathogens and intensive care units representing the epicenters of this crisis. Our findings are consistent with and contribute to the growing body of evidence documenting substantial AMR rates within the MENA region.^2,3,5^

Our finding of 45.0% ceftriaxone resistance in E. coli (95% CI 40.5–49.5) is markedly higher than the 34.5% reported in a recent Qatari meta-analysis,^4^ signaling a pressing local problem that directly challenges the ongoing efficacy of this agent as first-line empirical therapy for serious community- onset infections in our context. The 50.0% ciprofloxacin resistance in E. coli corroborates elevated fluoroquinolone resistance in the region,^9,10^ severely limiting oral therapeutic options.

The 30.0% MRSA rate underscores the persistent challenge of this pathogen, aligning with burdens reported in other GCC countries and necessitating reinforced decolonization and infection control strategies.^5,11^

The most concerning findings pertain to carbapenem resistance. The 55.0% meropenem resistance rate among ICU K. pneumoniae isolates signals a potential crisis, often restricting treatment to older, more toxic agents like colistin. This suggests an escalation beyond pre-pandemic levels reported from Qatar.^5^ The preserved susceptibility of CRKP to colistin, while currently reassuring, underscores our dependency on this last-line agent and mandates strict stewardship to preempt the emergence of colistin resistance.^12^ The significantly higher prevalence of MDROs in ICUs (45.0%) compared to wards (25.0%, p < 0.001) underscores this unit’s role as an amplification hub, driven by intense antimicrobial pressure, invasive devices, and high patient acuity.^9,13^

The variation in resistance by specimen source is noteworthy. The high resistance in urine-derived E. coli mirrors both community AMR pressure and prior antibiotic exposure in our patient population. Conversely, the different profile of wound isolates, dominated by S. aureus, reflects the distinct ecology of surgical and trauma-related infections. This underscores the need for specimen-specific empirical guidelines.

Furthermore, the substantial resistance in E. coli from the emergency department mirrors the community’s AMR reservoir, directly impacting the efficacy of empirical therapy for severe community-onset infections.^10^ This calls for an integrated “One Health” approach to AMR surveillance in Qatar, encompassing human, animal, and environmental data to fully comprehend and mitigate resistance drivers.^14^

The primary strength of this study lies in its comprehensive, contemporary baseline data, which provides immediate, actionable intelligence for our institutional ASP and Infection Prevention and Control committees.

This study has several limitations. Its retrospective, single-center design provides a robust prevalence snapshot but cannot assess temporal trends or guarantee broad generalizability to other settings in Qatar, highlighting the need for future prospective, multi-center surveillance. Secondly, the absence of molecular data [e.g., for Extended-Spectrum Beta-Lactamase (ESBL), carbapenem genes] limits our understanding of the underlying resistance mechanisms and transmission dynamics. Third, as a laboratory-based study, we lacked linked clinical outcome data such as mortality, length of stay (LOS), or detailed prior antibiotic exposure, which limits our ability to assess the clinical impact of the resistance patterns we observed. A key immediate research priority is to integrate whole-genome sequencing into our surveillance to elucidate these mechanisms and guide precise, targeted interventions.

## 5. CONCLUSION

In conclusion, this 2024 baseline analysis demonstrates a high burden of antimicrobial resistance at our institution, with critical care settings associated with the highest rates. The high rates of resistance to both first-line and last-resort antibiotics are associated with critical care settings and necessitate a multifaceted response. Our results support the urgent revision of empirical antibiotic guidelines, the reinforcement of infection prevention and control protocols especially in ICUs, and the intensification of antimicrobial stewardship with a focused effort on optimizing the use of high-risk agents. To effectively curb the AMR threat and safeguard patient outcomes, future initiatives must incorporate molecular epidemiology and establish a robust, prospective, multi-center surveillance network across Qatar.

## ACKNOWLEDGEMENT

The author(s) have no acknowledgements to declare.

## AUTHORS’ CONTRIBUTIONS

BA and ANA designed the study and developed the methodology. JA, AMA, and MH were responsible for data collection and curation. MB performed the formal statistical analysis. HA contributed to methodology development and data interpretation. BA supervised the entire research project and provided project administration. All authors contributed to the writing of the original draft, critically reviewed and revised the manuscript, and have read and approved the final version for publication.

## CONFLICT OF INTEREST

All authors declare that they have no conflict of interests.

## DISCLOSURE OF AI USE

The authors acknowledge the use of OpenAI’s language model and Grammarly for assistance with language editing, grammar refinement, and readability improvement during the preparation of this manuscript.

## DATA AVAILABILITY

The data that support the findings of this study are available from the corresponding author upon reasonable request.

## FUNDING

The author(s) received no financial support for the research, authorship, and/or publication of this article.

## ETHICAL APPROVAL

This study was approved by the Institutional Review Board of the Medical Research Center at Hamad Medical Corporation (Approval number: MRC-01-25-331). The requirement for informed consent was waived due to the retrospective design of the study.

## Figures and Tables

**Table 1.** Baseline demographic and clinical characteristics of the study population (N = 1,218 patients, 1,656 samples).

Characteristic	Value
**Total Patients**	1,218
**Total Samples**	1,656
**Mean Age (years ± SD)**	52 ± 16.5
**Gender, *n* (%)**	
Male	950 (78.0)
Female	268 (22.0)
**Specimen Type, *n* (%)**	
Urine	745 (45.0)
Blood	497 (30.0)
Wound Swabs	414 (25.0)

**Table 2.** Distribution of predominant bacterial pathogens by specimen type.

Pathogen	Blood (*n* = 497) *n* (%)	Urine (*n* = 745) *n* (%)	Wound (*n* = 414) *n* (%)	Total (*n* = 1656) *n* (%)*
*Escherichia coli*	75 (15.1)	410 (55.0)	41 (9.9)	526 (31.8)
*Staphylococcus aureus*	99 (19.9)	75 (10.1)	166 (40.1)	340 (20.5)
*Pseudomonas aeruginosa*	99 (19.9)	75 (10.1)	83 (20.0)	257 (15.5)
*Klebsiella pneumoniae*	75 (15.1)	112 (15.0)	21 (5.1)	208 (12.6)
Other	149 (30.0)	73 (9.8)	103 (24.9)	325 (19.6)

*Percentages are calculated based on the total number of isolates per specimen type (i.e., column percentages) and represent the proportion of each pathogen among isolates obtained from that specimen source. Totals may not sum to 100% due to rounding.

**Table 3.** Antimicrobial resistance profiles of predominant pathogens*.

Pathogen (*n*)	Ceftriaxone % (95% CI)	Ciprofloxacin % (95% CI)	MRSA % (95% CI)	Meropenem % (95% CI)	Ceftazidime % (95% CI)	ICU-Specific Resistance
*E. coli* (495)	45.0 (40.5–49.5)	50.0 (45.5–54.5)	–	–	–	–
*S. aureus* (414)	–	–	30.0 (25.6–34.7)	–	–	–
*K. pneumoniae* (166)	40.0 (32.6–47.7)	–	–	12.0 (7.5–18.0)^†^	–	ICU: 55.0% meropenem-R
*P. aeruginosa* (248)	–	35.0 (29.2–41.1)	–	–	28.0 (22.6–34.0)^‡^	ICU: 60.0% ceftazidime-R

*Antibiotics selected represent K. pneumoniae; classes: ceftriaxone (3rd-generation cephalosporin) and ciprofloxacin (fluoroquinolone) are first-line agents; MRSA is a critical resistance phenotype; meropenem (carbapenem) and ceftazidime (anti-pseudomonal cephalosporin) are last-line agents for Gram-negative infections*; ^†^Overall meropenem resistance in K. pneumoniae; ^‡^Overall ceftazidime resistance in P. aeruginosa.
